# RESULTS FROM A 2019 SURVEY OF STATE AGENTS ON ASSISTED LIVING REGULATIONS AND TRENDS

**DOI:** 10.1093/geroni/igz038.2007

**Published:** 2019-11-08

**Authors:** Brian P Kaskie, Portia Cornell, Paula Carder, Kali Thomas

**Affiliations:** 1 University of Iowa, College of Public Health, Iowa City, Iowa, United States; 2 Brown University, Providence, Rhode Island, United States; 3 OHSU-PSU School of Public Health, Porland, Oregon, United States

## Abstract

AL is regulated at the state level. Yet, little is known about the structure and function of state agencies that license and monitor AL. We fielded a 21-question survey among state agents with responsibility for AL in all 50 states. While licensure definitions of AL vary, state efforts appear uniform in regard to administrative alignment with departments of health as well as roles with facility licensing, renewal, and monitoring. However, we observed variability in the approaches used to monitor AL. While 80% of agents reported being able to issue fines for failures to meet regulatory standards, only 40% of states collected information concerning individual resident status. Only 20% issue separate licenses for providing care to persons with dementia, whereas 30% of state agents affirmed that non-licensed AL facilities were operating within their state. We consider how these varied regulatory approaches may shape facility operations and impact resident outcomes.

